# Factors related to the success of smoking cessation: A retrospective cohort study in Korea

**DOI:** 10.18332/tid/144272

**Published:** 2022-02-08

**Authors:** Yoon Hee Eum, Ho Jun Kim, Seolah Bak, Sung-Ha Lee, Jinri Kim, Su Hyeon Park, Seo Eun Hwang, Bumjo Oh

**Affiliations:** 1 Department of Family Medicine, Seoul Metropolitan Government-Seoul National University Boramae Medical Center, Seoul, Republic of Korea; 2 Department of Family Medicine, Hongseong Medical Center, Chungcheongnam-do, Republic of Korea; 3 Center for Happiness Studies, Seoul National University, Seoul, Republic of Korea; 4 Department of Family Medicine, H+ Yangji Hospital, Seoul, Republic of Korea

**Keywords:** predictor, nicotine dependence, smoking cessation, Fagerström score, underlying disease

## Abstract

**INTRODUCTION:**

Every year, at least half of the smokers in South Korea attempt to quit smoking. However, the Korean smoking rate remains still high among OECD countries. This study aimed to identify the factors that influence the success of smoking cessation efforts.

**METHODS:**

The study included 1395 smokers, who participated in a 12-week program comprising doctor counseling and pharmacological treatment (i.e. varenicline), conducted at smoking cessation clinics in two general hospitals from 2015 to 2019. The participants responded to a survey questionnaire inquiring about their smoking behaviors at the first visit to the clinic. After completing the program, they were asked whether they succeeded in smoking cessation. Based on participants’ reported success or failure, multivariable logistic regression analyses were conducted to obtain adjusted odds ratios (AORs) and 95% confidence intervals (CIs) for factors related to smoking cessation success.

**RESULTS:**

Following the 12-week program, 39.6% of the participants (n=553) succeeded in smoking cessation. Lower rates of nicotine dependence (AOR=0.73; 95% Cl: 0.54–0.98) and lower total amounts of smoking (AOR=0.67; 95% Cl: 0.47–0.95) were significantly associated with higher success rates in smoking cessation. In addition, smokers who participated in the program for at least 8 weeks (AOR=7.16; 95% Cl: 5.57–9.20) and smokers who had hypertension (AOR=1.40; 95% Cl: 1.07–1.85) or a cardiovascular disease (AOR=1.68; 95% Cl: 1.03–2.75) achieved higher success rates.

**CONCLUSIONS:**

Smokers’ success in smoking cessation was influenced by the period of visits to the smoking cessation clinic, the severity of nicotine dependence, and the presence of a cardiovascular disease including hypertension. Using these factors, smoking cessation strategy may be improved and personalized for individuals.

## INTRODUCTION

As of 2012, over 58000 people have died of smoking-related diseases in South Korea. The leading cause of smoking-related deaths was lung cancer, followed by stroke and ischemic heart disease^1^. Smoking is a major risk factor in premature death, and increased attention has been given to developing strategies for smoking cessation to reduce related deaths. In fact, smoking cessation at any age can prolong an individual’s lifespan; and among smokers aged 25–34 years, it can extend their lifespan by 10 years^2^.

In South Korea, the smoking rate of men aged >15 years was 31.6% in 2017, ranking second after Turkey (40.1%) among OECD countries^3^. According to the National Health and Nutrition Survey in South Korea, the smoking rate of adults aged >19 years decreased from 30.2% in the early 2000s to 22.3% in 2017. The smoking rate of men decreased significantly from 60.9% in 2001 to 38.1% in 2017; however, the smoking rate of women slightly increased from 5.2% in 2001 to 6.0% in 2017^4^.

The South Korean National Health Insurance began providing financial support for smoking cessation treatment to all citizens seeking support in February 2015 through clinics and public health centers. Varenicline and bupropion are currently the primary pharmaceutical treatments used for smoking cessation, and varenicline has been widely used due to its efficacy and efficiency^5^. In a study conducted in Taiwan and Korea on the effect of varenicline on smoking cessation, up to 59.5% of patients succeeded in quit smoking after 12 weeks. Furthermore, the smoking cessation maintenance rate was 46.8%, 12 weeks after the end of treatment (compared to 21.8% in the placebo group)^6^. In a study conducted in Turkey in 2011 on the effect of bupropion and varenicline on smoking cessation among 405 smokers, the smoking abstinence rate was higher in varenicline group (13.9%) than in the bupropion group (6.2%) one year after treatment^7^.

Varenicline is currently the most used treatment in smoking cessation clinics in South Korea. However, despite the implementation of smoking cessation programs using varenicline, and an annual budget dedicated to smoking cessation clinics, the smoking rate in South Korea has not decreased as expected^8^. Therefore, it is necessary to investigate the factors that impact smoking cessation success, such as smoking habits and health-related risks, in addition to pharmaceutical treatment. In this study, we compared the nicotine dependence and health-related characteristics of smokers who succeeded in smoking cessation and those who did not succeed, using a retrospective approach.

## METHODS

### Study design and participants

Participants were recruited among smokers who visited the smoking cessation clinics of two hospitals in Seoul between 2 March 2015 and 31 December 2019. All participants were aged >19 years and agreed to participate in the smoking cessation program. During the program, they received counseling from a medical professional (i.e. a family physician) and were prescribed smoking cessation medication up to 6 times over a period of 8 to 12 weeks.

**Figure 1:**
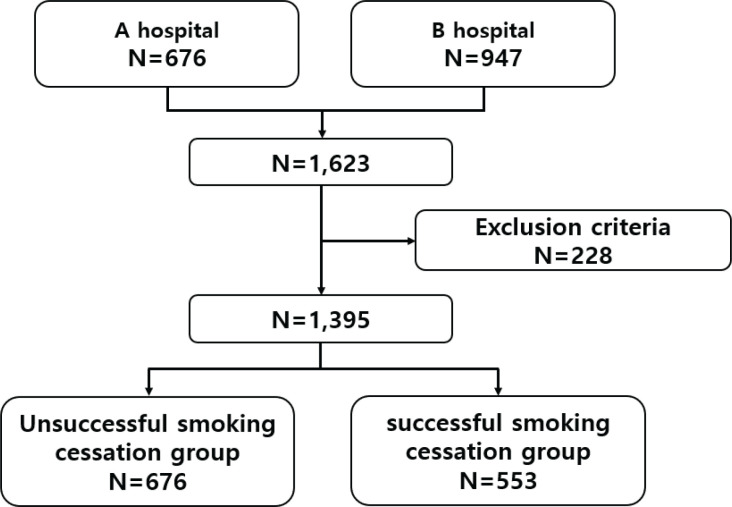
Study sample and flowchart of selection of participants

On their first visit to the clinic, participants were asked to complete a survey questionnaire on basic information and their smoking behaviors. After 12 weeks of counseling and varenicline treatment, the success of participants’ smoking cessation efforts was evaluated. Of the 1623 participants who took varenicline, 1395 were included in the final analysis; 228 participants who did not respond to the survey items necessary for analysis were excluded (Figure 1).

### Data collection and analysis

*Survey questionnaire*

The initial survey questionnaire included general information questions on gender, date of birth, height, and weight, and questions on nicotine dependence using the Fagerström test for nicotine dependence (FTND)^9^; amount of alcohol consumed (per instance and per week); smoking habits (e.g. amount of smoking, duration of smoking); pharmaceutical treatments taken and underlying chronic diseases; and previous attempts to quit smoking, duration of smoking cessation, and reasons for previous failures of smoking cessation. Body mass index (BMI, kg/m^2^) values of <25 were considered normal weight and >25 overweight/obese. Based on the Korean guidelines^10^, the following recommended amounts are considered moderate drinking: ≤8 drinks/week for men aged <65 years; ≤4 drinks/week for men aged >65 years and women aged <65 years; and ≤2 drinks/week for women aged >65 years.

*Determining smoking cessation success*

Participants were divided into successful and unsuccessful smoking cessation groups. Success in smoking cessation was determined based on participants’ report that they had successfully quit smoking on their last visit to the clinic after the 12-week program. Participants who discontinued their visits in the middle of the program (i.e. low compliance) were included in the group who failed to quit smoking.

### Statistical analysis

Chi-squared test was used to test independence between the two groups (successful and unsuccessful cessation), and two group t-test was used to compare the means. Multivariable logistic regression analyses, according to smoking cessation success, were conducted to obtain adjusted odds ratio (AORs) and 95% confidence intervals (CIs) for factors related to the success of smoking cessation. Odds ratios were adjusted for sex, age, underlying diseases, and alcohol consumption. Stata ver. 15.0 was used in the data analyses. A p<0.05 was considered statistically significant.

## RESULTS

### Participants’ sociodemographic characteristics and smoking history

Of the 1395 participants, 553 (39.6%) succeeded in smoking cessation after a 12-week smoking cessation program. Regardless of success or failure to quit smoking, more than half were aged 40–64 years. More than 85% of the participants in both groups were male. The average smoking period (prior to participation in the program) of the successful cessation group was longer (29.75 years, SD=12.58, t-test p=0.001) than that of the unsuccessful cessation group (27.42 years, SD=13.46). In addition, the number of patients who visited the smoking cessation clinic for more than 8 weeks was higher in the successful group (n=405; 73.24%) than in the unsuccessful group (n=241; 28.62%) (chi-squared test, p<0.001). The average nicotine dependence score (based on the FTND) in the successful group was lower (4.63) than the average score in the unsuccessful group (5.01) (t-test p=0.004). The presence of underlying chronic diseases, including hypertension (p<0.001), cardiovascular disease (p=0.011), and cancer (p=0.040) was significantly higher in the successful smoking cessation group. There were no significant differences between the successful and unsuccessful smoking cessation groups in rates of obesity or high-risk alcohol consumption (Table 1).

**Table 1:** Descriptive characteristics of participants by reported success in smoking cessation, South Korea (N=1395)

	*Unsuccessful smoking cessation n (%)*	*Successful smoking cessation n (%)*	*p*
**Total**, n	842	553	
**Age** (years)			
19–39	233 (27.67)	89 (16.09)	<0.001
40–64	472 (56.06)	353 (63.83)	
≥65	137 (16.27)	111 (20.07)	
**Sex**			
Men	741 (88.00)	474 (85.71)	0.212
Women	101 (12.00)	79 (14.29)	
**BMI** (kg/m^2^)			
Normal	526 (62.47)	340 (61.48)	0.710
Obese	316 (37.53)	213 (38.52)	
**Smoking amount** (cigarettes/day), mean ± SD	17.98 ± 8.76	17.13 ± 8.81	0.078
**Years smoking,** mean ± SD	27.42 ± 13.46	29.75 ± 12.58	0.001
**Pack-years**			
<10	152 (18.05)	100 (18.08)	0.914
≥10 and <20	203 (24.11)	128 (23.15)	
≥20	487 (57.84)	325 (58.77)	
**Attempt to quit smoking last 1 year**			
Yes	352 (41.81)	254 (45.93)	0.128
No	490 (58.19)	299 (54.07)	
**Duration of smoking cessation** (months)			
<6	780 (92.64)	496 (89.69)	0.043
≥6 and <12	28 (3.33)	34 (6.15)	
≥12	34 (4.04)	23 (4.16)	
**Underlying diseases**			
Hypertension	192 (22.80)	177 (32.01)	< 0.001
Diabetes mellitus	120 (14.25)	97 (17.54)	0.097
Dyslipidemia	151 (17.93)	121 (21.88)	0.069
Pulmonary disease	37 (4.39)	17 (3.07)	0.211
Cardiovascular disease	37 (4.39)	42 (7.59)	0.011
Neurological disease	27 (3.21)	19 (3.44)	0.815
Neuropsychiatric disease	28 (3.33)	19 (3.44)	0.911
Cancer	2 (0.24)	6 (1.08)	0.040
**Visited the clinic for >8 weeks**	241 (28.62)	405 (73.24)	< 0.001
**Alcohol drinking**			
No	271 (32.19)	177 (32.01)	0.962
Moderate	288 (34.20)	193 (34.90)	
Problematic	283 (33.61)	183 (33.09)	
**FTND**			
Low (0–3)	239 (28.38)	188 (34.00)	0.082
Medium (4–6)	370 (43.94)	227 (41.05)	
High (7–10)	233 (27.67)	138 (24.95)	
**FTND score,** mean ± SD	5.01 ± 2.41	4.63 ± 2.46	0.004
**Fagerström questionnaire 1** How soon after you wake up do you smoke your first cigarette? (minutes)			
>60	92 (10.93)	102 (18.44)	0.001
31–60	148 (17.58)	89 (16.09)	
6–30	251 (29.81)	165 (29.84)	
≤5	351 (41.69)	197 (35.62)	
**Fagerström questionnaire 2** Do you find it difficult to refrain from smoking in a place where it is forbidden?			
Yes	289 (34.32)	164 (29.66)	0.069
No	553 (65.68)	389 (70.34)	
**Fagerström questionnaire 3** Which cigarette do you hate most to give up?			
All others	443 (52.61)	290 (52.44)	0.950
The first one in the morning	399 (47.39)	263 (47.56)	
**Fagerström questionnaire 4** How many cigarettes do you smoke?			
≤10	175 (20.78)	139 (25.14)	0.118
11–20	444 (52.73)	282 (50.99)	
21–30	146 (17.34)	96 (17.36)	
≥31	77 (9.14)	36 (6.51)	
**Fagerström questionnaire 5** Do you smoke more frequently during the first hours after waking than the rest of the day?			
Yes	366 (43.47)	241 (43.58)	0.967
No	476 (56.53)	312 (56.42)	
**Fagerström questionnaire 6** Do you smoke even if you are so ill that you are in bed most of the day?			
Yes	499 (59.26)	299 (54.07)	0.055
No	343 (40.74)	254 (45.93)	

FTND: Fagerstrom test for nicotine dependence. BMI: body mass index (kg/m^2^).

### Factors associated with smoking cessation success

Table 2 presents a comparison of the FTND scores on each item, duration of smoking, period of visits to the smoking cessation clinic, and underlying chronic diseases in each group using a multivariable logistic regression. The success rate of smoking cessation in the group with high FTND scores was 0.73 times lower than the group with low FTND scores (AOR=0.73; 95% CI: 0.54–0.98). In particular, the success rate of smoking cessation was significantly lower in all groups who smoked within 1 hour of waking up in the morning than the group who smoked 1 hour after waking up (AOR=0.58; 95% CI: 0.39–0.86, AOR=0.61; 95% CI: 0.43–0.87, AOR=0.52; 95% CI: 0.37–0.73). The success rate of the group who smoked ≥31 cigarettes per day was 0.57 times lower than the group who smoked ≤10 cigarettes per day (AOR=0.57; 95% CI: 0.35–0.91). The success rate of the group who smoked ≥20 pack-years was 0.67 times lower than the group who smoked <10 pack-years (AOR=0.67; 95% CI: 0.47–0.95). The success rate was 7.16 times higher among those who visited the clinic for ≥8 weeks than those who visited for <8 weeks (AOR=7.16; 95% CI: 5.57–9.20). The success rate increased 1.4 times for patients with hypertension (AOR=1.40; 95% CI: 1.07–1.85) and 1.68 times for patients with a cardiovascular disease (AOR=1.68; 95% CI: 1.03–2.75).

**Table 2:** Factors associated with smoking cessation rate (logistic regression) among South Korean smokers (N=1395)

	*OR (95% CI)*	*p*	AOR* (95% CI)	*p*
**FTND**				
Low (0–3) (Ref.)	1	0.044	1	0.032
Medium (4–6)	0.78 (0.61–1.00)		0.80 (0.62–1.04)	
High (7–10)	0.75 (0.57–1.00)		0.73 (0.54–0.98)	
**Fagerström questionnaire 1** How soon after you wake up do you smoke your first cigarette (minutes)?				
>60 (Ref.)	1	0.001	1	0.001
31–60	0.54 (0.37–0.79)		0.58 (0.39–0.86)	
6–30	0.59 (0.42–0.84)		0.61 (0.43–0.87)	
≤5	0.51 (0.36–0.71)		0.52 (0.37–0.73)	
**Fagerström questionnaire 2** Do you find it difficult to refrain from smoking in a place where it is forbidden?				
No (Ref.)	1	0.068	1	0.063
Yes	0.81 (0.64–1.02)		0.80 (0.63–1.01)	
**Fagerström questionnaire 3** Which cigarette do you hate most to give up?				
All others (Ref.)	1	0.950	1	0.876
The first one in the morning	1.01 (0.81–1.25)		0.98 (0.79–1.22)	
**Fagerström questionnaire 4** How many cigarettes do you smoke?				
≤10 (Ref.)	1	0.037	1	0.030
11–20	0.80 (0.61–1.05)		0.82 (0.62–1.09)	
21–30	0.83 (0.59–1.17)		0.82 (0.57–1.17)	
≥31	0.59 (0.37–0.93)		0.57 (0.35–0.91)	
**Fagerström questionnaire 5** Do you smoke more frequently during the first hours after waking than the rest of the day?				
No (Ref.)	1	0.967	1	0.636
Yes	1.00 (0.81–1.25)		0.95 (0.76–1.18)	
**Fagerström questionnaire 6** Do you smoke even if you are so ill that you are in bed most of the day?				
No (Ref.)	1	0.055	1	0.106
Yes	0.81 (0.65–1.00)		0.83 (0.67–1.04)	
**Clinic visit period** (weeks)				
<8 (Ref.)	1	<0.001	1	<0.001
≥8	6.82 (5.37–8.68)		7.16 (5.57–9.20)	
**Pack-years**				
<10 (Ref.)	1	0.832	1	0.017
≥10 and <20	0.96 (0.68–1.34)		0.84 (0.58–1.21)	
≥20	1.02 (0.76–1.36)		0.67 (0.47–0.95)	
**Underlying disease**				
**Hypertension**				
No (Ref.)	1	<0.001	1	0.016
Yes	1.59 (1.25–2.03)		1.40 (1.07–1.85)	
**Diabetes mellitus**				
No (Ref.)	1	0.098	1	0.682
Yes	1.28 (0.96–1.71)		1.07 (0.78–1.47)	
**Dyslipidemia**				
No (Ref.)	1	0.069	1	0.636
Yes	1.28 (0.98–1.68)		0.93 (0.68–1.26)	
**Pulmonary disease**				
No (Ref.)	1	0.214	1	0.138
Yes	0.69 (0.38–1.24)		0.63 (0.34–1.16)	
**Cardiovascular disease**				
No (Ref.)	1	0.012	1	0.037
Yes	1.79 (1.13–2.82)		1.68 (1.03–2.75)	
**Neurological disease**				
No (Ref.)	1	0.815	1	0.648
Yes	1.07 (0.59–1.95)		0.86 (0.46–1.62)	
**Neuropsychiatric disease**				
No (Ref.)	1	0.911	1	0.941
Yes	1.03 (0.57–1.87)		1.02 (0.55–1.90)	
**Cancer**				
No (Ref.)	1	0.062	1	0.067
Yes	4.61 (0.93–22.91)		4.63 (0.90–23.93)	

* AOR: adjusted odds ratio; adjusted for sex, age, underlying diseases, and alcohol consumption.

FTND: Fagerström test for nicotine dependence.

## DISCUSSION

Results of this study show that the smoking cessation success rates were higher among smokers with lower FTND scores, who smoked 1 hour after waking up, visited the smoking cessation clinic for more than 8 weeks, and smoked a lower total amount of cigarettes (pack-years). Further, the success rate of smoking cessation among participants with hypertension and cardiovascular disease was higher than among participants without the abovementioned diseases.

Smoking cessation support services are available nationwide in South Korea through various channels, such as smoking cessation clinics, smoking cessation counseling service lines, and websites that provide information about smoking cessation^11^. Such efforts to reduce smoking have been recognized worldwide, and South Korea has achieved high levels of performance in the tobacco control policies included in the Framework Convention on Tobacco Control (FCTC).

Although several evaluations of smoking cessation programs have been conducted nationally, poor data fidelity may contribute to the lack of research on factors related to successful smoking cessation. To obtain reliable data, we identified the success factors of smoking cessation among more than 1300 adults who wished to quit smoking.

A study of 90 patients at a smoking cessation clinic in a public hospital in 2014 found that religion affected the success of smoking cessation. Also, it showed that the higher the patient’s dependence on nicotine before participating in the cessation program, the lower the probability of cessation success^12^. Results of the present study are similar in terms of dependence, but as the study questionnaire did not include inquiries about religion, its effect on smoking cessation success could not be evaluated. Another study investigated the factors affecting success in smoking cessation among 114 patients who visited a smoking cessation clinic in a hospital from 1998 to 2000 and who received cessation treatment using a nicotine patch and nortriptyline. In the successful smoking cessation group, the participants’ age and body mass index (BMI) were significantly higher while their exhaled carbon monoxide concentration measured at the initial visit to the clinic was significantly lower than participants in the unsuccessful smoking cessation group. Also, it showed that nicotine dependence scores (determined using the FTND) of the successful smoking cessation group were lower but statistically insignificant^13^. In the current study, we found a similar inverse relationship between nicotine dependence and smoking cessation success rates; however, there was no relationship between obesity and cessation success rates. Consequently, personalized smoking cessation strategy is necessary according to the FTND score before an attempt to quit smoking.

In a study of 54 patients, who took varenicline in a smoking cessation clinic from 2016 to 2017, the success rate of smoking cessation among depressed patients reduced to 0.201 times compared to patients who were not depressed. This result was based on the evaluation of depression using the BDI scale^14^. In the current study, only 47 patients responded that they had a psychiatric disease in the questionnaire, as such no meaningful results were obtained in regard to depression. Psychological factors are important for success in smoking cessation, therefore if the current study included a large number of patients with psychiatric disorders, there would also be significant results.

A study comparing 2000 current smokers and former smokers using data from the Korean National Health and Nutrition Survey showed that the higher the number of cigarettes smoked per day, the higher the smoking cessation success rate^15^. In contrast, the current study, as well as some other previous studies, showed that the higher the amount of smoking, the lower the success rate^16,17^. The latter may be explained by the assumption that increase in total amount of smoking (pack-years) is linked with higher nicotine dependence^18^, which results in lower success rate of smoking cessation. Another hypothesis is that the smoking cessation clinic is free, which may have affected the motivation of smoking cessation participants, resulting in different results.

Our study revealed that patients with hypertension or cardiovascular disease had a higher rate of smoking cessation success than participants without such diseases. A cross-sectional study of adult men admitted to hospitals for cardiovascular disease in 2004 explored the patients’ perception on health benefits of smoking cessation and their willingness to quit smoking; 78% of patients believed that smoking cessation will reduce heart disease, 93% answered that they were willing to quit smoking, and 88% replied that they preferred to quit smoking without any help^19^. Since the study was conducted before the implementation of a national smoking cessation support project, patients’ awareness of the relationship between cardiovascular disease and smoking would probably improve if the same study was conducted today. Thus, patients with the abovementioned disease have a higher awareness compared to their healthy counterparts. Similarly, such patients were exposed to doctor’s explanations about the necessity of quitting smoking, and were referred to smoking cessation clinics by their physicians, which may have had a significant impact on the success rates of smoking cessation. On the other hand, even though pulmonary disease is strongly correlated with smoking, there was no significant difference in the success rate of smoking cessation. Rather it seems that the success rate of smoking cessation is decreasing when there are pulmonary diseases. Previous findings showed similar results, and maybe due to the high nicotine dependence of COPD patients^20^. As such, it may be helpful if doctors more actively explain the necessity of smoking cessation to patients who visit the hospital for hypertension or for cardiovascular disease or for other diseases as well.

Several local studies investigating the predictors of smoking cessation have shown mixed results depending on the study method or on the number of participants. The current study is a retrospective cohort study with a sample size of more than 1000 smokers who took varenicline – which is the most commonly used pharmacological treatment in smoking cessation clinics – and compared the survey results of two groups (successful and unsuccessful) after completing a smoking cessation program. Thus, the current study could complement the mixed results of previous studies.

### Limitations

The study is not without limitations. First, the number of participants with psychiatric conditions and cancer was small, so no meaningful results in this regard could be confirmed. Further research is necessary to include participants with these underlying conditions. Second, since success in smoking cessation was determined based on participants’ report at their last visit to the clinic, the self-reported success rate may be unreliable. It would have been preferable to use exhaled carbon monoxide concentrations to assess success rates. Third, only patient factors were considered and analyzed; non-patient factors, such as hospital location and doctors’ counseling techniques, were not included. Fourth, the study did not investigate whether participants returned to smoking after completing the smoking cessation program. A follow-up study on the successful smoking cessation group over a period of time is recommended. Fifth, one of the important factors for success in quitting smoking is motivation, but the current study did not analyze the motivation for smoking cessation. A more elaborately designed follow-up study may identify variables necessary for success in smoking cessation and enhance personalized smoking cessation programs.

## CONCLUSIONS

The smokers’ success in smoking cessation was influenced by the period of visits to the smoking cessation clinic, the severity of nicotine dependence, and the presence of hypertension or a cardiovascular disease. Using these factors to predict success of smoking cessation may improve personalized smoking cessation strategy for individuals. Further study on the factors on success of smoking cessation is necessary.

## Data Availability

The data supporting this research are available from the authors on reasonable request.
